# Aid conditionalities, international Good Manufacturing Practice standards and local production rights: a case study of local production in Nepal

**DOI:** 10.1186/s12992-015-0110-3

**Published:** 2015-06-14

**Authors:** Petra Brhlikova, Ian Harper, Madhusudan Subedi, Samita Bhattarai, Nabin Rawal, Allyson M. Pollock

**Affiliations:** Centre for Primary Care and Public Health, Queen Mary University of London, 58 Turner Street, London, E1 2AB UK; School of Social and Political Science, University of Edinburgh, 15a George Square, Edinburgh, EH8 9LD UK; Patan Academy of Health Sciences, Nepal and Central Department of Sociology and Anthropology, Tribhuvan University, Kathmandu, Nepal; Northeastern University, Boston, MA USA; Central Department of Sociology/Anthropology, Tribhuvan University, Kathmandu, Nepal

**Keywords:** Nepal, Local pharmaceutical production, Good manufacturing practice (GMP), Harmonisation

## Abstract

**Background:**

Local pharmaceutical production has been endorsed by the WHO as a means of addressing health priorities of developing countries. However, local producers of essential medicines must comply with international pharmaceutical standards in order to be eligible to compete in donor tenders. These standards determine production rights for on-patent and off-patent medicines, and guide international procurement of medicines. We reviewed the literature on the impact of Good Manufacturing Practice (GMP) on local production; a gap analysis from the literature review indicated a need for further research. Over sixty interviews were conducted with people involved in the Nepali pharmaceutical production and distribution chain from 2006 to 2009 on the GMP areas of relevance: regulatory capacity, staffing, funding and training, resourcing of GMP, inspectors’ interpretation of the rules and compliance.

**Results:**

Although Nepal producers have increased their overall share of the domestic market, only the public manufacturer, Royal Drugs, focuses on medicines for public health programmes; private producers engage mainly in brand competition for private markets, not essential medicines. Nepali regulators and producers state that implementation of GMP standards is hindered by low regulatory capacity, insufficient training of staff in the industry, financial constraints and lack of investment for upgrading capital. The transition period to mandatory compliance with WHO GMP rules is lengthy. Less than half of private producers had WHO GMP in 2013. Producers are not directly affected by international harmonisation of standards as they do not export medicines and the Nepali regulator does not enforce the WHO standards strictly. Without an international GMP certificate they cannot tender for donor dependent health programmes.

**Conclusions:**

In Nepal, local private manufacturers focus mainly on brand competition for private consumption not essential medicines, the government preferentially procures essential medicines from the only public producer while donor funded programmes rely on international manufacturers compliant with international GMP standards. We also found evidence of private hospitals bypassing national medicines approvals process.

Policies in support of local pharmaceutical production in developing countries as a source of essential medicines need to examine carefully how GMP regulations impact on regulators, local industry and production of essential medicines in practice.

## Introduction

The *Global strategy and plan for action on public health, innovation and intellectual property* adopted by the 61st WHA in 2008 identified local pharmaceutical production as one of the key building blocks required for needs-based essential health research and development addressing health priorities of developing countries [1]. Initiatives calling for local pharmaceutical production are not new. Local production has been on the WHO’s agenda since 1978 and in the 1970s and 1980s governments, UN and other international organisations supported the development of local pharmaceutical production in many low-income countries on the grounds that it would promote self-sufficiency in the medicine supply; reduce dependency on imports and improve foreign trade balances; and create employment opportunities [2–4]. Although questions were raised about economic feasibility and product quality [5] many developing countries succeeded in building a viable pharmaceutical industry satisfying more than 70 % of their national requirements of essential medicines [6, 7]. A robust evidence base on the positive impact of local production on access to medicines is limited and mixed, with the majority of studies focusing on the affordability of local products compared to their imported counterparts [8]. In a recent study, the locally produced essential medicines in Tanzania showed an overall higher availability across the country over imported products, which were more likely to be available in urban areas [9].

Post TRIPS, the strategic importance of local production was again brought forward as a way to benefit from public health flexibilities specified in the Doha Declaration [4]. However, the viability of local production depends on several factors including effective regulation, funding, technology transfer, economies of scale, and procurement policies. The procurement of essential medicines, especially for donor funded programmes, has been accompanied by a shift from national governments to international public-private partnerships so that local producers must comply with international pharmaceutical standards if they are to be eligible for tenders.

This paper is particularly concerned with GMP (Good Manufacturing Practice) which is a requirement for international procurement of medicines for donor and publicly funded health programmes. Although compliance with GMP is a condition of market entry, as well as conferring production rights for many essential medicines procured though international donors, there has been little analysis of the impact of international GMP standards on local production for public health systems and government use.

This in itself is of concern as the growth in international pharmaceutical standards and the process of their harmonisation, specifically through the International Conference on Harmonisation (ICH), has been criticised for raising standards and compliance costs with insufficient attention being paid to whether they improve research on medicines and the safety of end products [10–12]. The WHO has warned that the standard setting process of the ICH is dominated by Western governments and their research-based pharmaceutical industry, describing the process as exclusive, non-consultative and lacking in local knowledge [13]. It also noted that the standards are too high for local production. Closures of small and medium producers as well as public vaccine producers due to stricter GMP requirements have been observed, for instance, in India [14].

Despite the WHO’s criticisms and the importance of the GMP standards for the availability of affordable and high-quality medicines and viability of local pharmaceutical production it is not clear from the literature to what extent local production in low- and middle-income countries has been affected by the international harmonisation of the regulations. In this paper we provide a brief background to GMP reported studies on the impact of GMP on local production before using the case study of Nepal to explore GMP implementation and enforcement from regulators’ and producers’ perspective in the context of development aid and aid conditionalities.

### Background to GMP standards and their international harmonisation

According to the WHO, GMP “is that part of quality assurance which ensures that products are consistently produced and controlled to the quality standards appropriate to their intended use and as required by marketing authorization” [15]. They apply to producers and other parties involved in labelling and packaging pharmaceutical products. GMP standards are intended to reduce the risks in production process including cross-contamination and mix-ups (e.g. confusions because of false labelling). GMP guidelines represent minimal standards that are a necessary condition for marketing authorization.

The WHO prepared its first version of GMP in 1967 at the request of the Twentieth World Health Assembly [15], but these are not legally binding on member states. The WHO has made clear that the implementation and enforcement of GMP is a matter for individual states and their respective drug regulatory bodies which can specify different sets of requirements.

In 1975 the WHO attempted to implement the rules at the global level through the Action Program on Essential Drugs and the Certification Scheme on the Quality of Pharmaceutical Products Moving in International Commerce. In the Certification Scheme, exporting countries certify domestic pharmaceutical companies as manufacturers of drugs authorized for the domestic market and that their compliance with the WHO GMP is checked on a regular basis [16].

Western industry, quite separately established its own standard setting initiative. The European Free Trade Association (EFTA) introduced a Pharmaceutical Inspection Convention (PIC) in 1970 and complemented it with Pharmaceutical Inspection Co-operation Scheme in 1995 with the aim of developing and harmonising GMP and inspection standards and to promote cooperation among participating authorities including exchange of information and experience. 44 EFTA and non-EFTA countries are participating in PIC/S [17].

The most recent and prominent harmonisation effort via the International Conference on Harmonization (ICH) is led by pharmaceutical regulators and industry representatives of the EU, Japan, and the US, and accepted by other developed countries. In 1999, the ICH brought *GMPs for Active Pharmaceutical Ingredients,* which apply to production of APIs for on-patent and off-patent medicines in ICH signatory countries and in other countries such as Australia and Canada. These guidelines were also adopted by PIC/S in 2001 and, despite the earlier criticism of ICH by WHO, formed the basis for the 2010 WHO GMP guidelines for APIs [18]. ICH standards are thus of growing importance in policing and regulating the global pharmaceutical industry and none more so than GMP. Given their global impact it is worth noting several features of the ICH harmonisation process for its implications for market consolidation and developing countries:

i) *Industry driven harmonisation of regulation*
  In 2000, the WHO warned that the ICH represented interests of drug regulatory authorities and representatives of pharmaceutical industry of 17 high-income countries; the negotiation processes excluded nongovernmental organizations, patient and consumer groups and lacked consultations with academics and medical practitioners; and “the additional safety benefits from these rigorous standards have not yet been demonstrated but the costs incurred by manufacturers in meeting these requirements are significant” [13]. The WHO representing developing countries has only observer status on ICH.
ii) *GMP enforcement in developing countries*
  Regulatory authorities in developing countries have been under increasing pressure to adjust their domestic regulatory systems in response to various international political issues. Whether it be WHO or ICH or FDA GMP standards, their implementation and enforcement is not a low cost exercise, as pharmaceutical companies and governments must develop capacity to implement and enforce the regulation. Although most sub-Saharan Africa countries have a legal basis for medicine registration, guidelines and assessment procedures, regulatory authorities have limited economic and human resources, and the enforcement of regulations is often discretionary so that many public and most local manufacturers do not meet WHO standards [19].
  The enforcement of GMP standards, however, is not just a problem for low- and middle-income countries. Recent safety concerns over medicines and APIs imported by developed countries illustrated difficulties of regulatory agencies to monitor production facilities of foreign suppliers [20].
iii) * medicine procurement and aid conditionalities*
  GMP standards are enforced through government drug procurement systems at international, national, and local level and in particular through aid conditions. International procurement agents including GDF and UNOPS require compliance with WHO GMP standards as a necessary condition for participation in a tender and may impose other conditions. Donors may also have their own requirements: the Global Fund requires compliance with WHO GMP ensured by the WHO Prequalification Programme and/or compliance with the US or EU rules [21]; PEPFAR requires US FDA GMP standards [22].
  These conditions may lock out local companies from their domestic market. For instance, producers in Tanzania complying with Tanzanian GMP guidelines have access to government tenders but cannot participate in more profitable international tenders requesting international GMP standards [2] and may be unable to tender for donor contracts within the country. Ugandan producers argue that the increased donor funding towards essential medicines combined with the requirement of WHO-GMP compliance means that “they are slowly being pushed out of the local essential medicines market” and some suggest that this “might be the single biggest threat to the survival of local pharmaceutical manufacturing” [23].

Another factor that inhibits local producers is size. Large MNCs argued for the size criterion to be included as a necessary condition for participation in international tenders. Their key argument is that small companies are not reliable in that they will not be able to supply drugs as promised [24].

Survival of local pharmaceutical production under international GMP rules ultimately depends on strength of drug regulation and access to sufficiently large markets. Stricter rules and related compliance costs need to be associated with additional safeguards ensuring medicine safety, efficacy and quality to be accepted by producers and, perhaps more importantly, in line with the access to affordable high quality medicines agenda. Trust in regulatory authorities and processes is a necessary condition for the rules to be accepted by purchasers of pharmaceuticals [25] thus securing demand which in turn provides incentives for producers to adopt the rules.

### Impact of GMP on local production

Our PubMed search identified only three studies on the issues of GMP in relation to local pharmaceutical production in low- and middle-income countries; these concerned the production of vaccines [26], antiretrovirals [27], and anti-malarials [28]. All three studies emphasize the need for local producers to invest in their production facilities and comply with GMP. The two public vaccine producers in Iran found it difficult to keep up with changing GMP requirements and although they satisfy the local needs, the informants identified the need for WHO prequalification and insufficient capacity as the key barriers to the export [26]. Abdo-Rabbo et al. examined the quality of animalarials at various levels of the distribution chain in Yemen and concluded that the problems with quality were not limited to a specific level of distribution chain and concerned locally produced as well as imported medicines [28]. None of the identified studies assessed the impact of GMP and their international harmonisation on local production.

A limited search of grey literature to check for additional studies and projects yielded two reports by MSF/DND Working Group which were concerned with the impact of ICH standards on the development and availability of medicines in developing countries [29, 30]. A comparison with the ICH and WHO GMP requirements concluded that although there were differences between the two sets of standards, they were ‘often marginal or formal’. The report further noted that ‘the main difference between ICH and WHO specifications is the interpretation of data submitted by applicants and the enforcement by DRAs’ [30].

In addition to regulatory capacity building, institutions such as UNIDO, WHO (Prequalification Project), and DfID support specific local pharmaceutical producers of, primarily, antiretrovirals, anti-TB and antimalarials in achieving WHO prequalification [31–33]. As of April 2015 only three out of 419 WHO prequalified medicine products were produced by a low-income country producer (119 HIC, 297 MIC) and none of prequalified APIs were produced in a low-income country (3 in HICs, 75 in MICs) [34, 35].

### Background to pharmaceutical production and regulation in Nepal

Allopathic medicine in Nepal has a relatively recent history. The limited supply of medicines to Nepal was via India, and the British Embassy for the elite (Interview, Kathmandu University, April 2007) until the first “people’s movement” of 1950. The more systematic development of the health sector began with increasing development aid assistance and the country’s roll over five year plans. Nepal government started to manufacture its own drugs in government facilities from the 1950s, focusing initially on medicinal plants and herbal forms and was located under the Ministry of Forests. The Royal Drugs Laboratory was set up as a pilot production site in 1965, and then converted to Royal Drugs Limited (RDL) in 1972 - the first production unit in Nepal (Interview, APPON, December 2006). The first private company, Chemidrug Industries Pvt. Ltd. was opened in 1971 (Interview, Kathmandu University, April 2007). The drug Act of 1978 resulted in the Department of Drug Administration (DDA) being set up in 1979 (where it was still part of the Ministry of Forests). Precursors to the Drug Act included the Black Marketing and other Social Offences Act, 2032 BS (1975), and the Drug Abuse Control Act, 2033 BS (1976) (see [36] for a full list of all the Acts pertaining to health, and their development in Nepal). By 1979 there were two Nepalese companies but around 1000 Indian ones; Nepal was an extension of the Indian market. It was not till after the late 1980s, however, that the nascent Nepalese industry started to mushroom. Relocated to a part of the Ministry of Health and Population, the DDA has overseen this growth of the Nepal pharmaceutical industry to its size of 58 registered companies in July 2014 (personal communication), and been responsible for the regulation of the industry.

Nepali pharmaceutical companies focus on the secondary and tertiary production (formulation and packaging) and supply medicines only to the local market. They had a 25-27 % share of the Nepali pharmaceutical market in 2004 while the import was dominated by Indian pharmaceutical producers (170 Indian companies out of nearly 250 importers) [37]. In interviews, Nepali producers talked of the 30:70 split in the market between Nepali and Indian products, and their aims of reversing this percentage. For 2006–2009 the share of local manufacture increased to 40 %; with domestic producers mainly catering to rural areas, where they have about 80 % of the market, and having about 20 % of the urban market [38].

In Nepal, although there is local production of some essential medicines, the majority are imported. Of the 537 products in various strengths and dosage forms listed on the 2011 National List of Essential Medicines, Nepali companies were producing less than one third, 176 products [38]. Fifteen drugs accounted for just over half (52 %) of local production in 2005 [37]. Of these eight were listed on the national essential medicines list and included amoxicillin (the highest selling drug), ciprofloxacin, iron preparations, paracetamol and metronidazole. The public manufacturer, Royal Drugs, focuses on medicines for public health programmes while the private producers engage mainly in brand competition in the private market [39].

The DDA is located in Kathmandu, and aims to “make available safe, efficacious and quality drug to the general public by controlling the production, marketing, distribution, sale, export–import, storage and use of drugs” through the selection of essential medicines and support of pharmaceutical industries to comply with WHO-GMP and to “achieve self-reliance in the production of essential drugs” [40]. (For a comprehensive overview of regulations in place see [41].)

## Methods

This paper uses a broadly ethnographic approach combining interviews, observations and a review of international and national literature collected within a broader research project ‘Tracing pharmaceuticals in South Asia’ [42]. In Nepal, from 2006 to 2009, the team undertook more than 170 transcribed semi-structured interviews, the majority of which were in Nepali and then translated into English. The interviewees were people involved in the pharmaceutical production and distribution chain including producers, medical representatives, pharmacists and providers with some having overlapping roles. Information was collected from four further categories: international donors, activists, regulators and scientists. The topics were based on an interview schedule and included the everyday working practices of the interviewees, with particular focus on the three main drugs of the research (rifampicin, fluoxetine, and oxytocin) and reflecting the broader issues of regulation, production, access to and rational use of medicines. In line with the iterative and reflexive qualitative research methodology, topics discussed reflected the empirical concerns and issues that emerged from the research. In addition extensive participant observation was conducted in a number of areas in and around Kathmandu, and in Western Nepal (in clinics, pharmacies, OPDs, with Medical Representatives on their rounds, and visits to two production plants.

Regulatory and policy documents were collected from the interviewees and from the websites of the Nepal medicine regulatory agency, WHO and donors.

Ethical review for the project ‘Tracing pharmaceuticals in South Asia’ as a whole was obtained from the School of Social and Political Science at the University of Edinburgh, and for the Nepali element from the Nepal Health Research Council (NHRC).

## Results

### Issues with GMP implementation in Nepal

- *Rules are subject to local discretion and regulatory capacity and not updated in line with WHO standards*

The rules and standards themselves are subject to a great deal of local discretion and interpretation and this in itself depends on the role of the enforcers and the staff who are employed.

Senior DDA officials told us they have developed regulations in the following areas: Drug registration regulations, Drug standard regulations, Drug Inquiry and Inspection Regulation and Drug manufacturing code.

Drug manufacturing code of 1984 is written in Nepali and published along with the WHO GMP code of practice (in English). Despite the WHO revising their GMP codes in 1998 and 2003, this part has not been updated in the DDA’s publication. We were told by a senior drug administrator that the DDA is in the process of publishing a new code as the 1984 DDA code does not explain certain things clearly; for example, it is written in the code that “fresh air” is necessary while producing drugs but it does not explain what is meant by this. When asked about overlap between the DDA code and WHO GMP code, he replied in vague terms, saying that most of the WHO GMP standards are incorporated in the DDA code (Interview, Senior Drug Administrator, DDA, June 2007).

From the 1990s the DDA made the upgrading of facilities to WHO GMP standards mandatory. The deadline was set for April 2007, but by the end of the data collection period in 2008 only eight companies had managed this. The then director of the DDA described that the WHO GMP certification for Nepali companies remained “optional”, with the DDA’s own Code on Manufacturing of Drugs the only legally binding requirement. As of 2013 26 of the 58 Nepali companies were compliant with the WHO GMP (personal communication, 2013).

GMP certification was described by the DDA as necessary only for export [43], although the WHO does inspect for drugs and products linked to their “own purposes” (for example vaccination programmes, TB drugs for DOTS, and ARVs). The WHO role is mainly indirect, through the DDA. While the Association of Pharmaceutical Producers of Nepal (APPON) are supposed to be assisting with this process, and doing trainings around GMP they are deemed by many to be of little help (as one company director stated: “they take our money and drink whisky”!). During the fieldwork period, they had a volunteer pharmacist from Japan helping them with this process of developing guidelines and trainings. APPON was more involved in lobbying for dollar rates for imports from India and for non-tariff barriers such as labelling of all foreign imports in Nepali.

b) *GMP training and capacity*

The Director of the DDA described the GMP certification process as part of the Essential Drugs and Medicines Programme. The DDA conducted the initial training in country, with support from the WHO which is “technical and financial”. However, the difficulties they face in implementing the GMP process were described to us as three fold. Firstly, regulatory capacity relating to the staff issues and their lack of expertise; this is not only DDA staffing problems (they had only five staff members who checked that rules were being followed), but the lack of expertise in the company staff. While there are increasing numbers of graduates now coming out of the universities, to date they have little experience. Pharmacy is a relatively new discipline in Nepal (Kathmandu University started their Pharmacist training course in 1994; the Institute of Medicine set up their School of Pharmaceutical and Biomedical Sciences in 1997; Pokhara University started in 2000; and Purbanchal University in 2005). By March 2014 there were 1400 pharmacy graduates registered with Nepal Pharmacy Council (personal communication) while the DDA register, in addition to the existing quality control laboratories and producers, reported 1688 wholesalers and 8800 retailers of allopathic medicines [44].

Secondly there are difficulties with understanding the concept. Some manufacturers say that they already sell well, so why do they need GMP? They have a “market perspective”, and as their drugs pass their own tests, why do they need it? They complain about the investments required for upgrading when they see little benefit. Thirdly, the GMP concepts themselves are changing and becoming more stringent.

c) *Regulatory requirements and associated costs*

An interview with a senior member of the of the quality control division of Nepal Drug Limited, the state run pharmaceutical company, which was struggling to remain in competition with the new private companies, reveals the issues they face with GMP (Interview notes, May 2007). He stated that they did not have the infrastructure to fulfil GMP standards; that the laboratory was not well equipped; there was not enough physical space; human resources were inadequate; there was no R&D budget; little administrative support; and that the location of the factory was wrong, due to the poor air quality in Kathmandu.

“If we have to go to GMP, we need the budget to improve some of the existing facilities, update them, and establish a new department to fulfil the requirement of WHO-GMP…. We have been discussing to hire consultants from outside to do feasibility study for focusing on IV Fluid (saline) WHO-GMP certificate”.

One group of senior management workers for one of the GMP certified companies described the sheer production of paperwork required for monitoring as overwhelming, besides the prohibitive costs. In addition, the director of one of Nepal’s largest pharmaceutical companies said that initially their production dropped after implementing GMP standards. They used to have “quality control”, but now this has shifted to “quality assurance” with greater stringency. This shift was described to us by another company’s senior manager as follows:

“Quality control is not in common use now. We call it quality assurance. Before while checking quality, they used to check at the end. But now they say that if we check it right from the beginning then quality is assured right from the beginning. The quality of excipient, whether the raw material is mixed properly or not, whether it is weighed properly or not, coating, punching, if everything is done properly, all this is checked. This is called SOP (Standard Operating Procedure)” (Interview, Kathmandu, April 2007).

One of the larger more established pharmaceutical companies had recently upgraded to GMP certification standards. The director of the company told us that the initial cost outlay had been 4 crore rupees (That is 40 million rupees, or a little over £300,000 at 2007 exchange rates). This had spun them from a profit making business into one with large debts. An ex-employee of Royal Drugs Nepal, stated that there was no way that this company could afford to upgrade to GMP standards. One particular complaint was that despite this initial outlay, the Nepal market was small and it would be difficult to recoup costs (the size of the Nepal market is stated to us a particular difficulty for Nepal to develop its own injectables; the market is just too small). Not one person we interviewed in the business thought of export as a possibility, and all were concentrating on the Nepal market.

A senior pharmacology professor referred to the problem in Nepal as one of quality versus cost. He referred to amoxicillin, which is now produced by nearly all the Nepali companies. It costs around 4–5 rupees, but if you find it for less than this then in his opinion the quality must be compromised. He reckoned that the then director at the DDA was good at his job, and working hard at trying to keep prices low while maintaining quality; he was working at trying to get GMP certification implemented. It was difficult, he said, as smaller companies used to send “goondas” around to him to ask why GMP was being put into place, claiming that it was driving up their costs and the prices of affordable medicines.

We were further told that the concepts themselves have changed a lot. An employee at one company explained to us how they had shifted to the AHU (air handling units) which are stricter, and of the use of “reverse osmosis” having replaced “demineralisers” for the water they use. The costs to run these new units had increased as well, and the size of the backup generators required to keep manufacturing standards up with the regular power cuts have increased. This was a particular problem in Kathmandu where during the time of the research power cuts of up to sixteen hours a day were frequent.

The GMP process was described to us by senior staff at one company where we were shown around the production site, and they bemoaned the sheer volume of recording necessary at every level of production. GMP certification considers many elements: the premises; personnel; quality control; production; sanitation and hygiene and finally, documentation. As they phrased it all the GMP process “should be done per documentation and documented”. Each and every activity is prescribed in detail through Standard Operating Procedures (SOPs), which are strategically displayed in Nepali and English throughout the site. The DDA was described as responsible for the guidelines that are set up for this end, and then responsible for their implantation (Interview, Kathmandu, April 2007).

d) *Impact on local industry and market*

Less than half of currently operating companies achieved the WHO-GMP certification, perhaps due to the rise in costs. Stricter measures taken in the case of imported products have resulted in some Indian companies being unable to import their products, and their products not being re-registered by the DDA (Interview, wholesaler, April 2007). The producer of “strepsils” (BOOTS) entered into a contract with a Nepali company to make this in Nepal, but because the company does not have GMP certification Strepsils are no longer available on the Nepal market.

#### Aid conditionalities and GMP

Although health expenditure from the general government has been steadily increasing the foreign aid remains a significant source of health care financing in Nepal (19 % of total health expenditure in 2008/09). The dependence is more pronounced in certain areas such as safe motherhood, immunisation, family health and planning, TB and leprosy control and STDs [45].

Government regulation currently states that any tender for the government procurement of drugs must be accompanied by the appropriate paperwork, which includes GMP certification. Despite the old state run Nepal Drugs (ND) not having GMP certification, contracts for public procurement are awarded as a priority to ND.

In the 1990’s ND was formulating rifampicin and supplying the National Tuberculosis Programme with about 1.1 million capsules annually (Interview, ND, February 2007). This was prior to the development of the WHO prequalification and the GDF), through which Nepal and other developing countries are currently supplied anti-TB drugs paid for by the Global Fund and other donors. After several years ND lost the contract and although it was not possible to ascertain the key reasons it was suggested that change in management and bribery caused the loss of contract (Interview, ND, February 2007). In addition, donors and conditions for international procurement of medicines changed. Currently, even with the capacity, ND would not be able to supply rifampicin to the national programme without WHO prequalification or other international GMP certificate.

During an interview with a senior advisor to the Ministry of Health and Population, we were told that the J-Vaccine (for Japanese Encephalitis) for Nepal was paid for by Japan and procured by the Nepal government from a company in China. The Chinese company was not GMP certified but the Japan government was happy with the quality and willing to give money. In 2007 the money for the vaccine was no longer given directly to Nepal but to UNICEF. Since the WHO, and other UN agencies, cannot procure any drugs without GMP certification the Chinese company, which had not applied for GMP by then, could not supply the product.

In 2005 MoHP launched a zinc programme in the public health sector procuring zinc from Nutriset in France. Local producers were also interested in supplying zinc. The POUNZ project introducing the zinc programme in the private sector assisted three local manufacturers with achieving the GMP audit and the three producers started supplying zinc in 2009. This would not be possible without the government commitment to this large scale programme and technical assistance offered by POUNZ. MoHP is planning to change procurement to the local suppliers who continued working with US Pharmacopeia representatives towards WHO prequalification with the view of reaching to international markets [46, 47]. As of May 2015 no Nepali manufacturer has achieved WHO prequalification [34].

These examples show some of the issues that arise from GMP certification in a heavily aid dependent state like Nepal.

#### Getting around registration: drugs and therapeutics committees?

Some (private) hospitals have established Drugs and Therapeutics Committees (DTCs) to get around market registration. One private hospital director explained that their committee – established in February 2006 - allows them to procure from any part of the world, even if that drug is not licensed in Nepal with the DDA. He explained that the DTC formation has been encouraged by the DDA who “don’t have physicians”, although the government says what the makeup of the committee should be. The DTC then can “import” medicines not registered, for example important drugs for their hospital cardiac medicines (the hospital specialised initially in cardiology – and has expanded from that). They have to produce “documents” – studies and outcomes from these drugs. Thus they are able to provide data for later registration.

A communication in the Kathmandu University Medical Journal suggested that these committees have a supportive function for the DDA:

“In developing countries like Nepal, where the pharmacovigilance programs are in its primitive stage, the DTC has immense responsibility in ensuring drug safety. This committee can also act as an advisory committee to the policy makers and drug regulatory authority of Nepal for drug safety matters based on their experiences” [48].

#### GMP and export

By 2012 no Nepali company had yet exported any pharmaceutical product [38]. However, GMP certification is also required for Ayurvedic products. Shakya documents the experience of a Nepali Ayurvedic company (Gorkha Ayurved Co.) with exporting medicinal herbs. The company had no idea that GMP certification was necessary (or that buyers could also ask for other internationally harmonised standard of Sanitary and Phytosanitary Measures (SPS). When the company set about the process of heading towards GMP certification, they found the RDRL (and the Department for Food Technology and Quality Control) “without any plan of policy regarding SPS standards, including GMP certification procedures”, particularly for Ayurvedic products [49]. Shakya is critical that the DDA had not prepared itself for accreditation processes, nor determined the basic mechanisms that companies should take. Businesses were pretty much on their own, suggested the author, with the company having huge outlays, including hiring a foreign expert to assist in the process.

It seems apparent that one consequence of attempting to harmonise its regulatory capacity will be a greater dependence on foreign assistance (both technical and financial) for this process.

## Discussion

Our research highlights several issues with implementation of international GMP standards in a country like Nepal and how international standards linked to foreign aid determine production rights of generic medicines where governments are dependent on aid. Domestic producers report that compliance with the stringent standards of GMP is a major obstacle for domestic production of affordable pharmaceutical products. The lenient approach to the enforcement of the GMP rules by the Nepali drug regulator, however, allows the growth of the industry which remains poorly regulated. These findings are relatively undocumented and unexplored in the literature.

The literature and our interviews highlight the issue of regulatory capacity building and the interpretation of standards by inspectors. International institutions and development agencies offer technical assistance to developing countries in the form of teaching GMP linked to capacity building [24, 50] yet, interviewees were concerned that since adherence to GMP standards is subject to individual experience and interpretation, inspectors from developed countries may impose more stringent rules than intended by regulators in developing countries (interviews with producers in India, [2]).

Our Nepali case study documents problems with GMP implementation and enforcement due to low regulatory capacity, insufficient training of staff in the pharmaceutical industry as well as financial constraints. Nepali producers do not produce APIs and target only the domestic market and to that extent they are not directly affected by internationally harmonised GMP requirements, but this also limits their market and their ability to recoup costs of investment in GMP related upgrades of manufacturing facilities. Nepali health programmes funded by international aid largely bypass government regulators and local producers as international agencies procure through large companies with an international GMP certificate. This impacts on local production through reduced economies of scale for some classes of medicines as Nepali companies do not have access to this part of the domestic market. This perhaps also explains the limited focus of the local industry on essential medicines.

Importance of local production capacity for the supply of essential medicines and availability of safe, efficacious and high quality medicines is high on the DDA’s agenda but there are few incentives for local manufacturers to produce essential medicines and the relatively long transition period before the compliance with WHO-GMP standards is mandatory challenges the acceptance of the stricter GMP requirements. Producers who already upgraded their production facilities have to absorb the cost of GMP compliance (resulting in higher prices of their products) without gaining recognition of high quality drug producers (as should be signalled by the GMP certificate). Some manufacturers question the need for GMP when their products sell well and there is no evidence to show that the GMP certificate guarantees high quality and GMP non-compliance results in low quality end products. It is, however, difficult to determine whether this is caused by weak enforcement or inadequacy of GMP standards in ensuring high quality of end products. These conditions are likely to perpetuate the ‘business’ practices of bonuses, gifts and substitution to push specific products as opposed to ‘ethical promotion’ [51].

## Conclusion

The Nepali local pharmaceutical industry has been growing significantly over the last decade. Many local producers are not affected by international quality standards as they do not export medicines and the DDA does not enforce the WHO GMP standards strictly. The evidence suggests that local private manufacturers focus on brand competition in private consumer markets and their production of essential medicines is limited to a few high-volume medicines as the government preferentially procures medicines from the only public producer and the donor funded health programmes rely on manufacturers compliant with international GMP standards.

Further research into access to affordable medicines via local pharmaceutical production in developing countries should consider the extent to which international standards assure quality, safety and efficacy and the capacities of national drug regulatory authorities to enforce standards where regulators have considerable interpretive license over standards and their implementation. As things stand, when aid conditionalities are linked to international standards the combined effect is to both determine and confer production rights and thus affect viability of local pharmaceutical industry and their incentives to produce essential medicines within low-income countries.
